# Disulfide proteomics of rice cultured cells in response to OsRacl and probenazole-related immune signaling pathway in rice

**DOI:** 10.1186/s12953-017-0115-3

**Published:** 2017-04-13

**Authors:** Kazuko Morino, Mayumi Kimizu, Masayuki Fujiwara

**Affiliations:** 1 National Agriculture and Food Research Organization, Central Region Agricultural Research Center, 1-2-1 Inada, Joetsu, Niigata 943-0193 Japan; 2 Keio University, Institute for Advanced Biosciences, 246-2 Mizukami, Kakuganji, Tsuruoka, Yamagata 997-0052 Japan

**Keywords:** Rice, Disulfide proteome, Monobromobimane, Reactive oxygen species, Os cold shock protein 2, Probenazole

## Abstract

**Background:**

Reactive oxygen species (ROS) production is an early event in the immune response of plants. ROS production affects the redox-based modification of cysteine residues in redox proteins, which contribute to protein functions such as enzymatic activity, protein-protein interactions, oligomerization, and intracellular localization. Thus, the sensitivity of cysteine residues to changes in the cellular redox status is critical to the immune response of plants.

**Methods:**

We used disulfide proteomics to identify immune response-related redox proteins. Total protein was extracted from rice cultured cells expressing constitutively active or dominant-negative OsRacl, which is a key regulator of the immune response in rice, and from rice cultured cells that were treated with probenazole, which is an activator of the plant immune response, in the presence of the thiol group-specific fluorescent probe monobromobimane (mBBr), which was a tag for reduced proteins in a differential display two-dimensional gel electrophoresis. The mBBr fluorescence was detected by using a charge-coupled device system, and total protein spots were detected using Coomassie brilliant blue staining. Both of the protein spots were analyzed by gel image software and identified using MS spectrometry. The possible disulfide bonds were identified using the disulfide bond prediction software. Subcellular localization and bimolecular fluorescence complementation analysis were performed in one of the identified proteins: *Oryza sativa* cold shock protein 2 (OsCSP2).

**Results:**

We identified seven proteins carrying potential redox-sensitive cysteine residues. Two proteins of them were oxidized in cultured cells expressing DN-OsRac1, which indicates that these two proteins would be inactivated through the inhibition of OsRac1 signaling pathway. One of the two oxidized proteins, OsCSP2, contains 197 amino acid residues and six cysteine residues. Site-directed mutagenesis of these cysteine residues revealed that a Cys^140^ mutation causes mislocalization of a green fluorescent protein fusion protein in the root cells of rice. Bimolecular fluorescence complementation analysis revealed that OsCSP2 is localized in the nucleus as a homo dimer in rice root cells.

**Conclusions:**

The findings of the study indicate that redox-sensitive cysteine modification would contribute to the immune response in rice.

**Electronic supplementary material:**

The online version of this article (doi:10.1186/s12953-017-0115-3) contains supplementary material, which is available to authorized users.

## Background

Reactive oxygen species (ROS) production is part of the early immune response in plants [1, 2]. Plants possess a layered defense system. PAMPs-triggered immunity (PTI), which induces calcium bursts, ROS generation, MAP kinase activation, salicylic acid (SA) and ethylene productions, defense-related gene expression, and callose deposition in the cell wall, represents one layer. Elicitor-triggered immunity (ETI), which is initiated by the interaction between pathogen-secreted molecules and plant receptors (so-called R proteins), constitutes the second layer. ETI causes local or hypersensitive response-like cell death and oxidative bursts [2]. In both layers, ROS is the principal signaling molecule. In rice, the small GTPase OsRac1 participates in ROS production by interacting with the respiratory burst oxidase homolog RbohB, producing ROS in a manner similar to human Rac1 in phagocytes [3, 4]. Furthermore, OsRac1 contributes to Pit (R protein)-mediated ETI and forms a protein complex with PTI-related proteins, such as HSP70, HSP90, Hop/Sti1a, OsRacGEF1, OsCERK1, and OsRACK1A [5, 6]. Of these, OsRACK1A is a ubiquitous WD40-repeat protein that is involved in various molecular processes [7]. In rice, OsRACK1A binds to the N terminus of NADPH oxidase, RAR1, or SGT1, which are the three key regulators of the immune response [5, 8]. Altogether, OsRac1 is a key regulator of both PTI and ETI and particularly of ROS production.

Probenazole (PBZ) is an activator of the plant immune response that functions in the SA signaling pathway [9, 10]. PBZ amplifies superoxide (O_2_^−^) production, a major ROS, by treating with an elicitor that was extracted from rice blast in the protoplast [9]. These observations indicate that PBZ modulates ROS production and activates PTI in rice.

During the immune response, ROS causes a drastic alteration in cellular redox status, which is sensed by redox-sensitive cysteine residues in redox proteins and is transmitted downstream [11, 12]. One such redox protein, NPR1, is an SA-responsive transcriptional co-activator in *Arabidopsis* that forms intermolecular disulfide bridges and presents as an oligomer in the cytoplasm. During the immune response, the changing cellular redox status induces the reduction of disulfide bridges in oligomeric NPR1 and the translocation of liberated monomeric NPR1 to the nucleus. In contrast, TGA, a transcription factor that interacts with NPR1, forms an intramolecular disulfide bridge, the reduction and subsequent modification of which cause its interaction with monomeric NPR1 in the nucleus, consequently activating the transcription of target genes [11, 13, 14]. Similarly, OsNPR1 functions in the SA signaling pathway, whose subcellular localization is controlled by the redox status in rice [10]. Recently, Xie et al. reported that cysteine residues in OsMAPK3 and OsMAPK6, which are reportedly involved in the immune response in rice, are sensitive to the redox status [12, 15]. These findings suggest that ROS production and accompanying redox modifications in cysteine residues play a critical role in the immune response in plants.

To identify redox proteins in plants, redox or disulfide proteomic approaches have been adopted [16–20]. These studies revealed that many proteins carry redox-sensitive cysteine residues and are regulated by the redox status in response to biological processes and environmental stresses. In a tomato, 90 potential redox proteins that respond to pathogen infection have been identified [20]. However, few potential redox proteins that are associated with the immune response have been detected in rice.

Using disulfide proteomic analysis, we attempted to identify potential redox proteins in rice cultured cells that respond to the activation or inhibition of the small GTPase OsRac1 or to PBZ treatment. We used the free thiol group-specific fluorescent probe monobromobimane (mBBr) to label reduced proteins in differential display two-dimensional gel electrophoresis (2-DE) [17]. Using this method, we were able to detect proteins carrying a reduced cysteine residue on the basis of the intensity of mBBr fluorescence. After the detection of mBBr fluorescence, the gel was stained with Coomassie brilliant blue (CBB), which is an indicator of total protein quantity (Additional file 1: Figure S1). We identified seven potential redox proteins, four of which responded to OsRac1signaling or PBZ treatment. Furthermore, we performed site-directed mutagenesis of potential redox-regulated cysteine residues in *O. sativa* cold shock protein 2 (OsCSP2), a potential redox protein that was identified by disulfide proteome analysis, and discovered that the Cys^140^ mutation leads to the mislocalization of proteins in rice root cells.

## Results

**Fig. 1:**
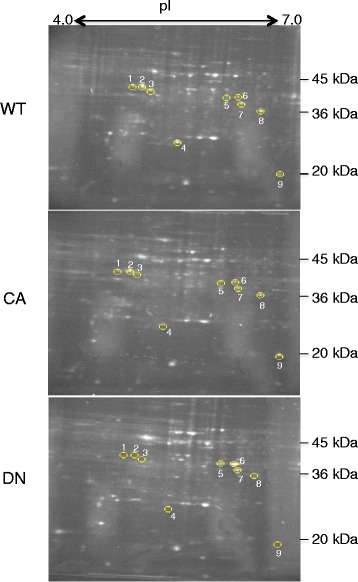
2-DE gel images of potential redox proteins tagged with mBBr. Gel images of mBBr-tagged proteins extracted from cultured rice (cv. *Nipponbare*) cells (WT; upper image), cultured cells expressing CA-OsRac1 (CA; middle image), and cultured cells expressing DN-OsRac1 (DN; lower image) in the presence of mBBr are shown. Potential redox proteins were marked and numbered. Their identities are shown in Table 1

**Fig. 2:**
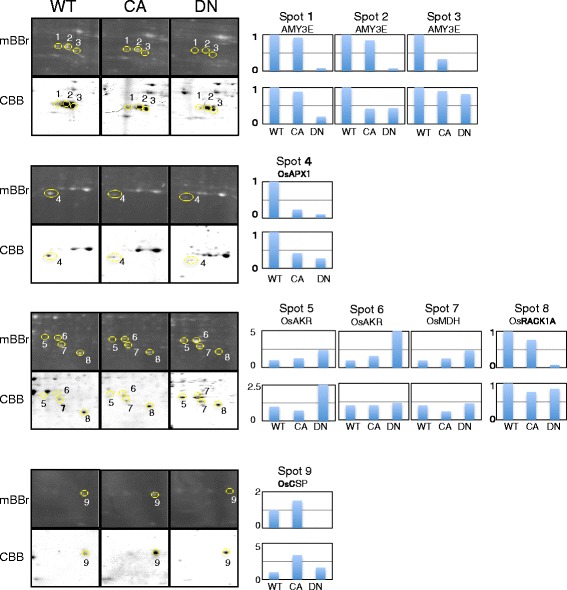
Magnified images of spots containing potential redox proteins labeled with mBBr and stained with CBB, and their relative intensity. Magnified mBBr-labeled protein spots in Fig. 1 (in upper images) and CBB stained identical spots (in lower images) are marked with circles and numbered. The relative intensity of the spots is shown in the bar graph on the right. The level in wild type cells is shown as 1. WT indicates wild type cultured cells; CA indicates cultured cells expressing CA-OsRac1; DN indicates cultured cells expressing DN-OsRac1

ROS production as a result of the activity of OsRac1, a key regulator of the immune response in rice, has been demonstrated in rice cultured cells [3]. Therefore, to identify a potential immune response redox protein, suspension culture cells were established from calli that was induced from mature non-transgenic plants (wild type) and transgenic plants expressing constitutively active (CA)- or dominant-negative (DN)-OsRac1. The redox status of these plants was evaluated in mature plants. ROS production was enhanced in plants expressing CA-OsRac1 or decreased in plants expressing DN-OsRac1 (Additional file 2: Figure S2). Total protein was extracted from non-transgenic rice and rice cultured cells expressing CA- or DN- OsRac1 in the presence of mBBr but no reducing agents. In rice cultured cells expressing CA-OsRac1, the signaling pathway downstream of OsRac1 is constitutively activated. Conversely, in rice cultured cells expressing DN-OsRac1, the signaling pathway downstream of OsRac1 is dominantly inhibited. Therefore, it is possible to detect proteins containing mBBr-tagged reduced cysteine residues that are regulated by the OsRac1 signaling pathway by comparing cells expressing CA- and DN-OsRac1 with wild type cells using 2-DE. The extracted proteins were separated by isoelectric focusing (pH 4.0–7.0) and run on a 12% sodium dodecyl sulfate polyacrylamide gel electrophoresis (SDS-PAGE) gel. The gel images of the spots containing mBBr-tagged reduced proteins that were visualized under ultraviolet (UV) light are shown in Fig. 1. We performed at least two biological replicates of 2-DE gel analysis and found nine protein spots that were different in mBBr-fluorescent intensity between wild type, CA-OsRac1, and DN-OsRac1 cells (Figs. 1 and 2).

To identify their constituent proteins, the nine spots were digested with trypsin in the gel and were analyzed by mass spectrometry and a data base search using the software Mascot (Matrix Science Ltd., London, UK; Table 1). Mass spectrometric analysis revealed that Spots 1, 2, and 3 contained the same protein alpha-amylase (isozymes AMY3E/AMY1.4). The intensity of

**Table 1:** Proteins carrying potential redox-sensitive cysteine residues in rice cultured cells expressing CA-OsRac1 or DN-OsRac1 or in PBZ-treated cultured cells

Spot Number^a^	Protein name	Accession Number	Gene Name	Identified peptide	Coverage (%)	MW^b^	pl^b^	Predicted disulfide bond^c^	Locus name
1	Alpha-amylase isozyme 3E	P27934	AMY3E/AMY1.4	3	12	45	4.769	9–109	Os08g0473600
2	Alpha-amylase isozyme 3E	P27934	AMY3E/AMY1.4	6	21.4	45	4.88	9–109	Os08g0473600
3	Alpha-amylase isozyme 3E	P27934	AMY3E/AMY1.4	6	12	44.3	4.975	9–109	Os08g0473600
4	L-Ascorbate Peroxidase 1, cytosolic	Q10N21	OsAPX1	3	22.8	28.34	4.26	32–168	Os03g0285700
5	Probable aldo-keto reductase 3	Q7XQ45	OsAKR	8	39	39.76	5.86	120–204	Os04g0339400
6	Probable aldo-keto reductase 3	Q7XQ45	OsAKR	8	35	39.29	6.04	120–204	Os04g0339400
7	Malate dehydrogenase, cytoplasmic	Q7XDC8	OsMDH	3	14.8	37.4	6.06	79–330	Os10g0478200
8	Receptor for activation C kinase 1A	P49027	OsRACK1A	10	24	35.6	6.23	150–203	Os01g0686800
9	Cold shock protein 2	Q84UR8	OsCSP2	3	25	20.3	6.46	140–150	Os08g0129200
10	Thioredoxin H1	Q0D840	OsTrxh1	7	45	10	5.19	11–43 40–43^d^	Os07g0186000
11	Receptor for activated C kinase 1A	P49027	OsRACK1A	8	46.7	35.8	6.33	150–203	Os01g0686800

^a^Spot Numbers are as given in Figs. 1 and 3

^b^Molecular mass and pI are as given in Figs. 1 and 3

^c^Predicted disulfide bonds were calculated using DiANNA1.1

^d^Disulfide bond was predicted based on similarities

mBBr and CBB were almost identical in Spot 1. The intensities of both mBBr and CBB in Spot 2 in DN-OsRac1 cells were decreased compared with those in wild type cells. The intensity of mBBr fluorescence in Spot 2 in CA-OsRac1 cells was not significantly decreased, whereas the intensity of CBB was apparently decreased compared with those in wild type cells. Therefore, AMY3E in Spot 2 was reduced in CA-OsRac1 cells and oxidized in DN-OsRac1 cells. mBBr fluorescence of Spot 3 in CA-OsRac1 cells was decreased, and could not be detected in DN-OsRac1, despite confirmation of the presence of protein by CBB staining. Therefore, AMY3E in Spot 3 was oxidized in CA- and DN-OsRac1 cells (Fig. 2).

Spot 4 contained *O. sativa* ascorbate peroxidase (OsAPX 1; Os03g0285700); Spots 5 and 6 contained the same protein, *O. sativa* aldo-keto reductase (OsAKR; Os04g0339400); Spot 7 contained *O. sativa* malate dehydrogenase (OsMDH; Os10g0478200). These enzymes are highly conserved among organisms and correlate with the redox status [21–25]. Although these three proteins were differently expressed in the wild type, CA-OsRac1, and DN-OsRac1 cells, the intensities of mBBr and CBB staining were approximately identical (Fig. 2). However, OsAPX1 (Spot 4) expression was decreased in both CA- and DN-OsRac1 cells compared with that in wild type cells, and OsAKR (Spots 5 and 6) and OsMDH (Spot 8) were increased in DN-OsRac1 cells (Fig. 2). Thus, although the OsRac1 signaling pathway is unrelated to the redox status of cysteine residues in OsAKR, OsAPX1, and OsMDH, it may regulate the expression levels of these proteins.

Spot 8 contained OsRACK1A, which is a reported effector of OsRac1 that is known to interact with OsRhohB [4, 5]. Spot 9 contained OsCSP2, which carries a cold shock domain and a zinc finger motif, and functions as an RNA and DNA chaperone under stress conditions [26, 27]. The mBBr fluorescence of Spots 8 and 9 were hardly detectable in DN-OsRac1 cells, despite confirming the presence of proteins by CBB staining. Therefore, OsRACK1A and OsCSP2 are oxidized when OsRac1 activity is inhibited.

**Fig. 3:**
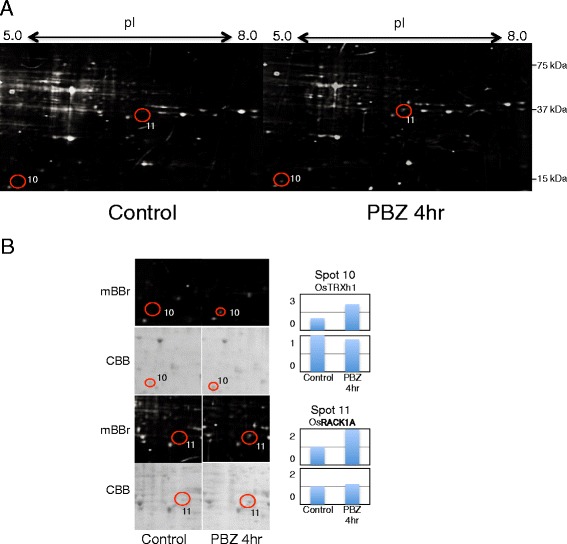
Potential redox proteins respond to PBZ treatment. **a** Proteins were extracted from rice (cv. *Nipponbare*) cultured cells (control; left images) and cultured cells treated with PBZ for 4 h (right images) in the presence of mBBr (upper images). Potential redox proteins were marked with circles and numbered. Their identities are shown in Table 1. **b** Magnified images of spots containing potential redox proteins labeled with mBBr and stained with CBB; their relative intensity is shown in the bar graph on the right. The level of intensity of the control is shown as 1

To identify redox proteins that were activated or modulated by PBZ, proteins extracted from cultured cells that were treated with PBZ were subjected to disulfide proteome analysis. We performed at least two biological replicates of 2-DE gel analysis, and representative gel images of mBBr-tagged protein spots are shown in Fig. 3a. Two protein spots were detected in PBZ- treated cultured cells that could not be detected in control cultured cells despite clear CBB staining for both PBZ treated and control cells (Fig. 3b). Therefore, these two spots consisted of proteins containing cysteine residues reduced by PBZ treatment. Mass spectrometry analysis revealed that Spot 10 contained *O.sativa* thioredoxin (OsTRXh1), a ubiquitous redox protein that exhibits disulfide oxidoreductase activity through the reversible oxidation of two cysteine residues in a CXXC motif to a disulfide [28, 29], whereas Spot 11 contained OsRACK1A, which was detected as an oxidized protein in cultured cells expressing DN-OsRac1 (Table 1). Taken together, we identified seven potential redox proteins.

To investigate whether the potential redox proteins that were identified contained intramolecular disulfide bonds, they were subjected to disulfide bond prediction analysis using the software DiANNA [19, 30]. We identified possible disulfide bonds between Cys^9^ and Cys^190^ in AMY3E, between Cys^32^ and Cys^168^ in OsAPX1, between Cys^120^ and Cys^204^ in OsAKR, between Cys^79^ and Cys^330^ in OsMDR, between Cys^150^ and Cys^203^ in OsRACK1A, and between Cys^11^ and Cys^43^ in OsTRXh1 (Table 1, Additional file 3: Figure S3). However, OsTRXh1 was predicted to have a reversible disulfide bond in its catalytic motif between Cys^40^ and Cys^43^ on the basis of its similarity to known orthologs [31]. OsRACK1A is a highly conserved scaffold protein among eukaryotes that interacts with various proteins [5, 7, 32, 33]. Although OsRACK1A carries eight cysteine residues, the presence of an intramolecular disulfide bond in OsRACK1A proteins has not been reported. On the basis of the results of disulfide bond prediction analysis, we found a possible intramolecular disulfide bond in OsRACK1A that is required to be confirmed in future studies (Additional file 3: Figure S3).

**Fig. 4:**
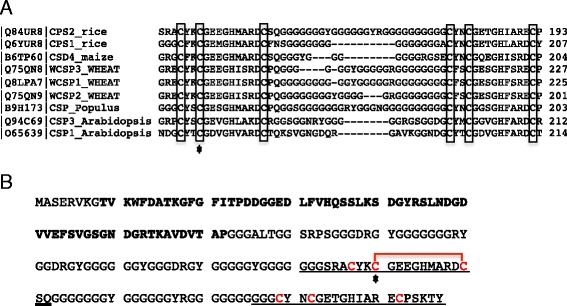
Potential redox-sensitive cysteines. **a** Alignment of the amino-acid sequences of cold shock proteins in plants. Conserved cysteine residues are shown in *boxes*. The *star* indicates Cys^140^. **b** Amino acid sequence of OsCSP 2. The cold shock domain is shown in *bold letters*; the zinc finger motifs are indicated by *underlined letters*; and cysteines are shown in *red*. The *star* indicates Cys^140^. The *red line* indicates a predicted disulfide bond

**Fig. 5:**
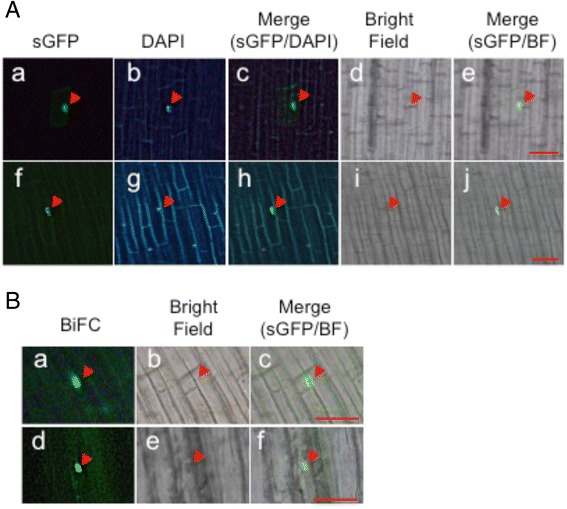
Subcellular localization of wild type and mutated OsCSP2 in the rice roots and BiFC analysis. **a** Subcellular localization of WtOsCSP2-sGFP fusion protein in a rice root cell (*a*). DAPI staining of (*a*) is shown in (*b*). A merged image of (*a*) and (*b*) is shown in (*c*). Bright field of (*a*) is in (*d*). A merged image of (*a*) and (*d*) is shown in (*e*). Mutated OsCSP2 (C140S)-sGFP showed abnormal localization in a rice root cell (*f*). DAPI staining of (*f*) is shown in (*g*). A merged image of (*f*) and (*g*) is shown in (*h*). Bright field of (*f*) is in (*i*). A merged image of (*f*) and (*i*) is shown in (*j*). *Arrowheads* indicate the localization of observed GFP signal. Scale bar represents 50 μm. **b** BiFC analysis of WtOsCSP2 is shown in (*a*). Bright field of (*a*) is in (*b*). A merged image of (*a*) and (*b*) is shown in (*c*). BiFC analysis of mutated OsCSP2 (C140S) is shown in (*d*). Bright field of (d) is in (*e*). A merged image of (*d*) and (*e*) is shown in (*f*). *Arrowheads* indicate the localization of observed BiFC (mKG) signal. Scale bar represents 50 μm

Of the potential redox proteins identified, we selected OsCSP2 to investigate the function of cysteine residues. OsCSP2 carries six cysteine residues in a zinc finger motif that are conserved among orthologs (Fig. 4a). A possible disulfide bond was found between Cys^140^ and Cys^150^ (Fig. 4b). The redox status of cysteine residues is known to be related to subcellular localization in numerous proteins [13, 34]. Therefore, we performed site-directed mutagenesis of six conserved cysteines to serines. Then, the wild type and mutated OsCSP2 proteins were fused to green fluorescent protein (sGFP) to observe their subcellular localization in rice root cells. The wild type and mutated OsCSP2-sGFP fusion proteins driven by the rice ubiquitin promoter were inserted into rice root cells via particle bombardment. After overnight incubation, sGFP expression was visualized. The wild type OsCSP2-sGFP protein was observed in the nucleus of rice root cells; however, OsCSP2-sGFP carrying the C140S mutation revealed an abnormal localization (Fig. 5A). The subcellular localization of OsCSP2 carrying a mutation in any other cysteine residue was the same as that of the wild type protein. The homo dimerization of chaperone proteins, which is mediated by redox-sensitive intramolecular disulfide bonds, has been reported in *Escherichia coli* Hsp33 [35]. Therefore, we conducted a bimolecular fluorescence complementation (BiFC) assay using split monomeric Kusabira Green (mKG; CoralHue® Fluo-Chase Kit; MBL International Corp., Woburn, MA, USA). We created constructs for BiFC for use with the particle bombardment method by fusing full-length wild type OsCSP2 or OsCSP2 carrying the C140S mutation to the C- or N-terminal fragments of mKG. The two constructs, with either the C- or the N-terminal fragments of mKG driven by the rice ubiquitin promoter, were bombarded into 5 day-old rice roots grown on 1/2 MS medium. After overnight incubation, BiFC fluorescence was observed. Similar fluorescence to that of the sGFP fusion protein shown in Fig. 5A was detected in cells that had been bombarded with OsCSP2-mKG_C and _N (Fig. 5 Ba). Therefore, wild type OsCSP2 forms a dimer, which predominantly localizes in the nucleus. Although we performed the same experiment using OsCSP2 carrying the C140S mutation, no clear difference from the wild type protein was evident (Fig. 5 Bd).

**Fig. 6:**
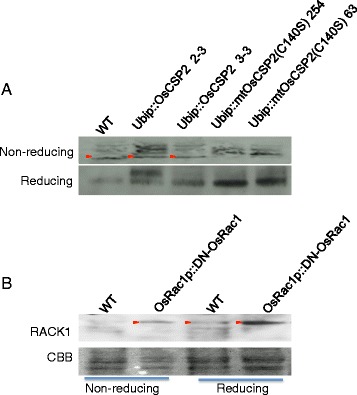
Western blotting under non-reducing and reducing conditions. **a** Detection of OsCSP2 in non-transgenic wild type (WT), transgenic plants over-expressing wild type OsCSP2 (line 2–3 and 3–3), and transgenic plants expressing mutated OsCSP2 (C140S) (line 254 and 63). *Red triangles* indicate specific bands for plants expressing wild type OsCSP2 under non-reducing conditions. **b** Detection of OsRACK1A in wild type cultured cells (WT) and cultured cells expressing DN-OsRac1 (*upper panel*). CBB staining of membrane is shown in the lower panel. *Red triangles* indicate the bands of the expected size

The disulfide bond formation was predicted between Cys^140^ and Cys^150^ in OsCSP2, and C140S mutation may be involved in subcellular localization but not its dimerization. Therefore, to confirm the occurrence of redox regulation at Cys^140^, we produced transgenic plants which over-expressing wild type OsCSP2 or OsCSP2 carrying the C140S mutation. Total proteins were extracted from a non-transgenic plant, two OsCSP2 overexpressing plants, and two plants expressing C140S OsCSP2, which were subjected to SDS-PAGE and Western blotting under reducing or non-reducing conditions. Under reducing conditions, the clear bands, which was expected size of CSP2 monomer from 2-DE analysis and the prediction of molecular weight were detected in all samples. However, under non-reducing conditions, multiple bands were detected in all samples; and their sizes differed among plants (Fig. 6a). Considering the results under reducing condition, multiple bands indicate the presence of different intracellular disulfide bonds in OsCSP2 among plants. The upper- and lowermost bands could not be detected in plants expressing C140S OsCSP2, suggesting that proteins in these bands would contain a disulfide bond involved in Cys^140^.

Next, to confirm the potential for redox sensitivity in OsRACK1A, Western blotting under reducing or non-reducing condition was performed using anti-OsRACK1A antiserum. We detected the band of the expected size in DN-OsRac1 under non-reducing conditions. However, this band of the expected size could not be detected in wild type cultured cells (Fig. 6b). Conversely, these bands could be detected in both cells under reducing conditions. Therefore, OsRACK1A in wild type cultured cells would interact with its interactor in non-reducing conditions, and this interaction would be inhibited with expression of DN-OsRac1.

## Discussion

The redox-based modification of cysteine residues is an important mechanism by which the structure, interactions, and subcellular localization of proteins are regulated [11, 12, 34, 35]. Numerous studies have demonstrated that ROS production during the immune response of plants is essential for both PTI and ETI [2]. The sensitivity of cysteine residues in proteins to the redox status is central to the immune signaling in plants. Through 2-DE of mBBr-labeled proteins and mass spectrometry, we identified seven potential redox proteins related to the OsRac1 signaling pathway or responsive to PBZ treatment.

One of the seven potential redox proteins identified was OsTRXh1, a well-known redox protein, which indicates that our approach was appropriate. OsTRXh1 is reportedly involved in the regulation of ROS production both during the development and in response to stress in rice [36]. We found that OsTRXh1 reduction increased after PBZ treatment in rice cultured cells (Fig. 3). This indicates that OsTRXh1 functions in controlling the redox status of proteins that participate in the PBZ-activated immune response.

Because OsAPX1, OsMDH, and OsARK were detected by mBBr labeling, they must carry reactive cysteine thiols. However, the intensities of mBBr fluorescence and CBB staining of the spots containing these proteins were approximately identical. Therefore, the redox status of these proteins cannot be regulated by the OsRac1 signaling pathway. In *Arabidopsis,* AtAPX1 (AT1G07890) and cytMDH1 (ATG04410) are listed as target proteins of Trx-h [37]. Hara et al. reported that cytMDH1 forms a homo dimer through a disulfide bond at Cys^330^ [38]. These studies imply that similar OsTRXh-related redox regulation, involving OsAPX1 and OsMDH exists in rice. ARKs consist of a large family of conserved enzymes that function in detoxification in response to multiple stresses, including ROS production and metabolic processes [22, 39]. In humans, ARK1B10 carries a cysteine residue key for enzymatic activity that is involved in thiol-disulfide exchange [23]. Although the function of the cysteine residues of plant ARKs has not been reported to date, we detected an OsARK by mBBr staining; thus, a free reactive cysteine residue must be present in OsARK in rice.

We identified Spots 1, 2, and 3 as containing alpha-amylase (AMY3E/AMY1.4; Table 1, Fig. 2). In *Arabidopsis,* chloroplast-targeted alpha-amylase AMY3 and plastid-targeted beta-amylase TR-BAMY are known to be redox proteins that are activated by reduction of thioredoxins [40, 41]. Cheng et al. reported that the gene expression of the alpha-amylase isozymes AY1A, AYB1, AYC2, AYA3D, and AY4A is inhibited in *O. sativa* lectin receptor-like kinase- knock out plants and actin-depolymerizing factor mutants, which exhibit the disruption of innate immunity [42]. Recently, it was reported that the expression of the alpha-amylase isozymes RAmy1A and Ramy3D is significantly decreased in plants in which OsRACK1A is downregulated [43]. These reports suggest that the regulation of several amylase isozymes is dependent on both the redox status and protein expression level. Although we were unable to distinguish between Spots 1, 2, and 3 in this study, the total expression of AMY3E was decreased in both CA- and DN-OsRac1 cells compared with that in wild type cells. AMY3E in Spot 2 was reduced in CA-OsRac1 cells but oxidized in DN-OsRac1 cells. In contrast, AMY3E in Spot 3 was oxidized in both CA- and DN-OsRac1 cells, yet the expression levels in these cells were similar to that in wild type cells (Fig. 2). Therefore, the OsRac1 signaling pathway must regulate both the redox status and expression level of AMY3E. It has been reported that AMY3E translocates to the plastid, a process dependent on its 25 amino acid signal peptide [44]. Because the contents of Spot 3 are smaller than those of Spots 1 and 2, one possibility is that Spot 3 contains oxidized AMY3E from which the signal peptide has been cleaved. Although sugar signaling is considered to play an important role in the immune response, the role of alpha-amylase in this process remains unclear [45]. Further studies are required to clarify the function and redox regulation of AMY3E during the immune response.

In our 2-DE analysis, OsRACK1A was oxidized in DN-OsRac1 cells and reduced in response to PBZ treatment. This suggests that the reactivity of the cysteine residues in OsRACK1A is increased by the PBZ-activated immune response and is suppressed by the inhibition of the OsRac1 signaling pathway. These results suggest that OsRACK1 A function as a sensor of redox status in rice immune signaling pathway through the oxidation-reduction reaction of its cysteine residues. However, we could not detect increasing reactive cysteine residues in cultured cells expressing CA-OsRac1 despite detection of increasing ROS production (Additional file 2: Figure S2). The overproduction of ROS may be toxic in cultured cells; therefore, it would not be feasible increase the reactive cysteine residues in cultured cells expressing CA-OsRac1.

In Western blotting, we detected the band of monomer size under non-reduced and reduced conditions in culture cells expressing DN-OsRac1. Conversely, this band could not be detected in wild type cultured cells under non-reducing conditions. Considering the results of 2-DE and Western blotting, OsRACK1A would carry intracellular disulfide bond and present as a monomer in culture cells expressing DN-OsRac1, although it may interact with its interactor by disulfide bond in wild type cultured cells (Fig. 6b). Although many studies have described OsRACK1A and its orthologs, the presence of intramolecular disulfide bonds has not been reported. We identified a predicted intramolecular disulfide bond, but detailed experimental evidence is required to confirm its existence. Recently, WD40-repeat proteins were reported to act as redox-sensitive proteins in response to pathogen infection in the resistant genotype of the tomato [20]. Therefore, the redox regulation of OsRACK1A in the rice immune response warrants further study.

OsCSP2 was also oxidized in DN-OsRac1 cells (Fig. 2). In the case of OsCSP2, the protein quantity was increased in cultured cells expressing CA- or DN- OsRac1 compared to the wild type, although reactive cysteine residues could be detected in cultured cells expressing CA- OsRac1 not in cultured cells expressing DN- OsRac1. This result indicated that OsCSP2 would be regulated by protein quantity and redox status through the OsRac1 signaling pathway. Six cysteine residues that are conserved among orthologs are present in OsCSP2, and a predicted intra molecular disulfide bond was found between Cys^140^ and Cys^150^ (Fig. 4b). The site-directed mutagenesis of these cysteine residues revealed that a Cys^140^ mutation causes abnormal subcellular localization of proteins in the root cells of rice (Fig. 5A). BiFC analysis determined that OsCSP2 forms a homo-dimer and localizes to the nucleus in rice root cells (Fig. 5B). However, we were unable to detect clear abnormalities or defects that were caused by the C140S mutation during BiFC. Therefore, we conclude that the redox status of Cys^140^ plays an important role in the subcellular localization of the monomer or other complexes but not in its homo dimerization and subcellular localization in the nucleus.

The results of Western blotting used with transgenic plants overexpressing wild type OsCSP2 or OsCSP2 carrying C140S mutation suggest that OsCSP2 could form multiple conformations depending on the intracellular disulfide bond at Cys140. (Fig. 6a). The functions of OsCSP2 and its orthologs have been reported; CSPs function as DNA or RNA chaperones under stress conditions [27, 46, 47]. Although the involvement of OsCSP2 in the immune response is unproven to date, cross talk clearly occurs between signaling pathways related to ROS signaling that respond to biotic and abiotic stress. Thus, further analysis of the function of OsCSP2 in the immune response is necessary.

## Conclusions

In conclusion, we performed disulfide proteomic analysis of mBBr-tagged proteins extracted from cultured rice cells expressing CA- or DN- OsRac1 and cultured cells that were treated with PBZ to identify immune response-related redox proteins. We identified seven potential redox proteins, which included the widely conserved redox protein, OsTRX1h1. Three potential redox proteins, AMY3E, OsRACK1A, and OsCSP2, appeared to be regulated by the OsRac1 signaling pathway, and OsTRXh1 and OsRACK1Awere reduced by treatment with PBZ. Site-directed mutagenesis of OsCSP2 revealed that Cys^140^, which is predicted to form a disulfide bond, participates in the subcellular localization of the monomer. These findings provide evidence that redox-sensitive cysteine modification plays an essential role in the immune response in rice.

## Methods

### Transgenic rice cultured cells and PBZ treatment

The promoter region of *OsRacl* was isolated by polymerase chain reaction using the primers 5′-tatacacggctaacctacacag-3′ and 5′-taggcggtgagaagaaacaagaac-3′. The isolated promoter sequence was then fused to *OsRac1*-G19V (yielding CA-OsRac1) or *OsRac 1-*T24N (yielding DN-OsRac 1) [3] in the binary vector pTN1 [48]. Transgenic plants expressing CA- and DN-OsRac1 were produced using *Agrobacterium-*mediated transformation with a G418-resistance marker driven by the *NCR* promoter as a selection marker [48]. Cultured cell suspensions were produced from rice cv. *Nipponbare calli* or transgenic plants expressing CA- or DN-OsRac1 and were maintained in an R2S medium [49] on a rotary shaker at 28 °C. For PBZ treatment, cells were pre-cultured in fresh medium overnight at 28 °C. One gram of cells was transferred to 1.5 ml of fresh medium containing 20 μM PBZ that was dissolved in dimethylformamide and was incubated for 4 h after treatment. As a control, cells were treated with dimethylformamide without PBZ.

### Protein extraction and mBBr labeling

Protein extraction and mBBr labeling were performed according to Yano et al. In brief, total protein was extracted from rice cultured cells by homogenization in Read yPrep™ Sequential Extraction Kit Reagent 1 (Bio-Rad Laboratories, Inc., Hercules, CA, USA) with 200 mM mBBr (AnaSpec, Inc., San Jose, CA, USA). The extract was purified using Ultracel® YM-10 protein concentrator (EMD Millipore Corp., Billerica, MA, USA), and the concentration was measured using the Bio-Rad Protein Assay (Bio-Rad Laboratories, Inc.).

### Protein separation by 2-DE

For the first dimension, 700 μg of protein in 185 μg of rehydration buffer was loaded on to an 11- cm IPG strip (pH 3–10, 4–7, or 5–8). Isoelectric focusing was performed with a protein isoelectric focusing cell (Bio-Rad Laboratories, Inc.), according to the manufacturer’s instructions. For the second dimension, SDS-PAGE was performed using 12% polyacrylamide gels. Gels were destained with a destaining buffer (10% acetic acid, 30% methanol) for 2 h, and then mBBr fluorescence were detected under UV light (365 nm) using a Fas charge-coupled device (CCD) system (Toyobo Co., Ltd., Tokyo, Japan) and a short-cut filter SC48 (Fujifilm Corp., Tokyo, Japan). After the detection of mBBr fluorescence, gels were stained with CBB G-250 (Bio-Rad Laboratories, Inc.) and visualized using a gel image analysis system (GE Healthcare, Little Chalfont, Buckinghamshire, UK).

### Image analysis and quantification

The patterns of spots on the 2-DE gels were analyzed using the gel image analysis software PDQuest™ (Bio-Rad Laboratories, Inc.) or Image Master 2D Platinum 6.0 (GE Healthcare).

### Mass spectrometry analysis

MS spectrometry analysis was performed according to the method described previously [12]. In brief, protein spots were digested with trypsin in the gel and analyzed by Q-TOF Ultima (Waters Corp., Milford, MA, USA). Peptide data were compared using the database software Mascot (Matrix Science Ltd.) to identify proteins.

### Bimolecular fluorescence complementation assay, the subcellular localization of sGFP fusion proteins, and transgenic plants

Full-length OsCSP2 cDNA was amplified from the rice cv. *Nipponbare* cDNA pool and cloned into the pCR™- Blunt II-TOPO® vector (Thermo Fisher Scientific, Waltham, MA, USA). Six clones of OsCSP2 carrying point mutations that substituted cysteine residues with serine residues were generated using PrimeSTAR® HS DNA Polymerase (Takara Bio Inc., Otsu, Shiga, Japan). The full-length wild type OsCSP2 and six mutant OsCSP2s were fused with sGFP or the C- or N-terminal fragments of mKG (CoralHue® Fluo-Chase Kit; MB International Corp.). The constructs were inserted downstream of the rice ubiquitin promoter in the vector pUC18. For producing transgenic plants, the wild type OsCSP2 cDNA and OsCSP2 carrying C140S, mtOsCSP2 (C140C), were fused with the rice ubiquitin promoter in PZH vector [50]. Transgenic plants expressing wild type CSP2 and OsCSP2 carrying C140S mutation were produced using *Agrobacterium-*mediated transformation with a hygromycin b resistance marker driven by the *CaMV35S* promoter as a selection marker. Plants were grown in a greenhouse under conventional conditions.

To visualize the subcellular localization of OsCSP2, the sGFP fusion constructs were introduced via the particle-bombardment method into 5 day-old rice roots that were grown on 1/2 MS medium (Biolistic® PDS-1000/He system; Bio-Rad Laboratories, Inc.). The roots were incubated in the dark for 16 h at 28 °C and then analyzed by fluorescence microscopy using a Leica DM4000B with an L5 filter (Leica Microsystems GmbH, Wetzlar, Germany). Fluorescent images were captured and processed using a cooled CCD camera (VB7000; Keyence Corp., Osaka, Japan). For the BiFC assay, the OsCSP2-C_mKG and OsCSP2-N_mKG fusion constructs were simultaneously inserted by particle bombardment into rice root cells, and fluorescence was visualized and imaged as described for sGFP.

### Western blotting under non-reducing and reducing conditions

Total proteins were extracted from rice culture cells or transgenic plants overexpressing wild- or mutated- OsCSP2 as described above. Proteins were mixed with reducing sample buffer containing 2-mercaptoethanol or non-reducing sample buffers and subjected to SDS/PAGE on 15% acrylamide gels. The proteins were blotted to PVDF membrane (Bio-Rad) using the manufacturer’s protocol. Membranes were probed with anti-OsRACK1A or anti-OsCSP2 antisera (1:1000 dilution) followed by detection with a goat anti-rabbit secondary antibody and chemiluminescent substrate (GE healthcare).

## Additional files

Additional file 1: Figure S1. 2-DE gel images of proteins. (PDF 642 kb)

Additional file 2: Figure S2. The presence of H2O2 and inhibition by NADPH oxidase inhibitor (Diphenyleneiodonium;DPI). (PDF 3745 kb)

Additional file 3: Figure S3. Predictive disulfide bond in potential redox proteins. (PDF 117 kb)

## Abbreviations

DPI: Diphenyleneiodonium sulfate
mBBr: monobromobimane
OsCSP2: *Oryza sativa* cold hock protein 2
PBZ: Probenazole
SA: Salicylic acid
