# Supplementary material for: Disulfide proteomics of rice cultured cells in response to OsRacl and probenazole-related immune signaling pathway in rice
Source: Proteome Sci. 2017 Apr 13;15:6. doi: 10.1186/s12953-017-0115-3 (PMC5390479; doi:10.1186/s12953-017-0115-3)
Supplement: Supplementary file 1 — 2-DE gel images of proteins. (PDF 642 kb) [file 12953_2017_115_MOESM1_ESM.pdf]

Supplementary Figure 1

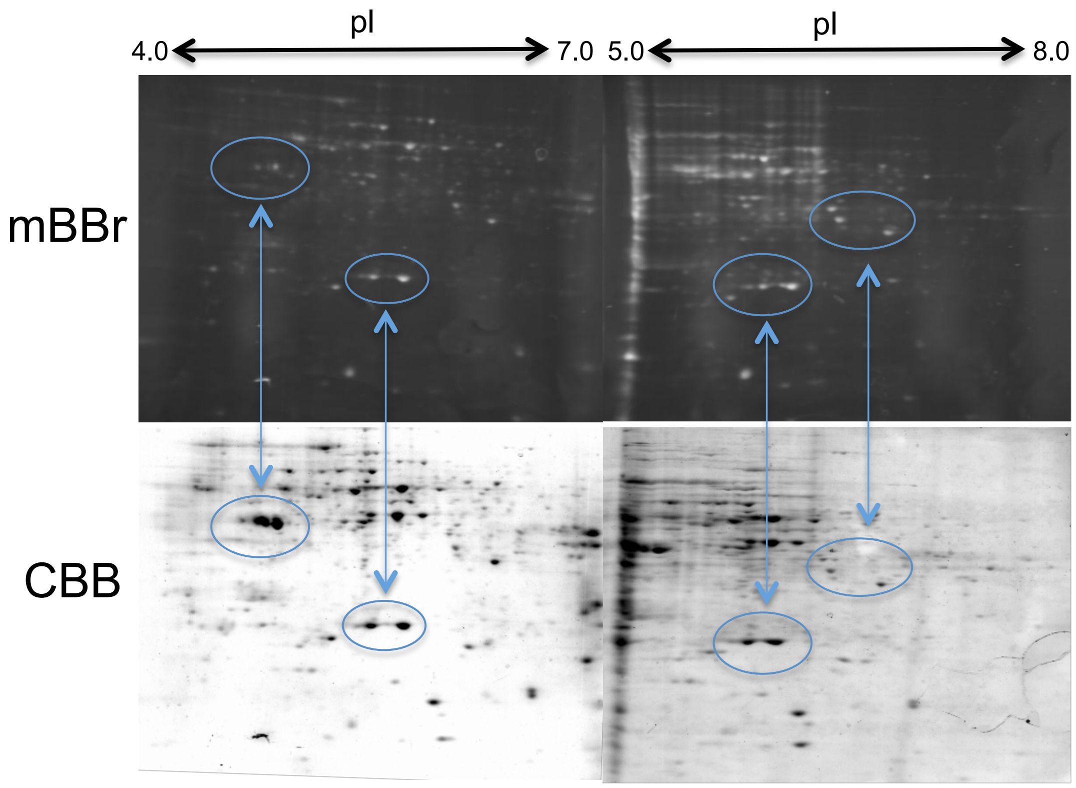

Supplementary Figure S1 2-DE gel images of proteins

The gel images of mBBr-tagged proteins which extracted from rice cv. Nipponbare cultured cells (Upper image) and of CBB staining proteins(Lower image).
In this method, proteins carrying a reduced cysteine residue can be detected by intensity of mBBr fluorescence. After detection of mBBr fluorescence, the gel was stained with Coomassie Brilliant Blue(CBB) which represents total protein quantity. Blue circles and arrows indicate identical spots between mBBr fluorescence and CBB staining. Isoelectric points were shown on top of photos.
