# Supplementary material for: Disulfide proteomics of rice cultured cells in response to OsRacl and probenazole-related immune signaling pathway in rice
Source: Proteome Sci. 2017 Apr 13;15:6. doi: 10.1186/s12953-017-0115-3 (PMC5390479; doi:10.1186/s12953-017-0115-3)
Supplement: Supplementary file 2 — The presence of H2O2 and inhibition by NADPH oxidase inhibitor (Diphenyleneiodonium;DPI). (PDF 3745 kb) [file 12953_2017_115_MOESM2_ESM.pdf]

Supplementary Figure 2

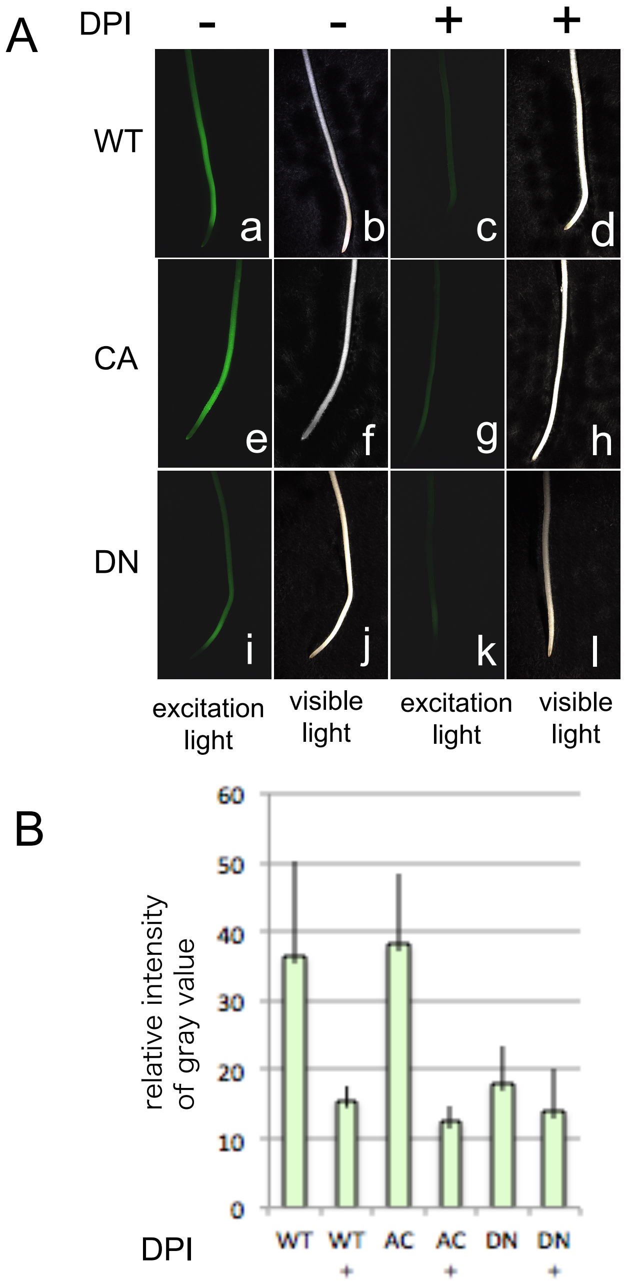

Supplementary Figure S2 The presence of H_2O_2 and inhibition by NADPH oxidase inhibitor (*Diphenyleneiodonium ;*DPI).

A. Five day-old non-transgenic cv. Nipponbare plants; WT (a, b, c, d), or with *CA-OsRac1*; CA (e, f, g, h), or with *DN-OsRac1*; DN (i, j, k. l). Seedlings treated with 20 µM DPI are shown in (c, d, g, h, k, l); untreated seedlings are shown in (a, b, e, f, i, j). DCFH-DA fluorescence was detected under excitation light and a GFP*2* filter; images in (a, c, e, g, i, k) are shown under visible light in (b, d, f, h, j, l), respectively. The + or the – on top of pictures indicate DPI - treated or - untreated, respectively. Using light for observations are indicated on bottom of photos.
B. Average of the relative fluorescent intensities (gray value) of photographs were measured used with imageJ and are shown. The error bars show S.E.
