# Supplementary material for: Disulfide proteomics of rice cultured cells in response to OsRacl and probenazole-related immune signaling pathway in rice
Source: Proteome Sci. 2017 Apr 13;15:6. doi: 10.1186/s12953-017-0115-3 (PMC5390479; doi:10.1186/s12953-017-0115-3)
Supplement: Supplementary file 3 — Predictive disulfide bond in potential redox proteins. (PDF 117 kb) [file 12953_2017_115_MOESM3_ESM.pdf]

Supplementary Figure 3

AMY3E

MGKHHVTLCC VVFAVLCLAS SLAQAQVLFQ GFNWESWRKQ GGWYNFLHEK
VEEIASTGAT HVWLPPPSHS VSPQGYMPGR LYDLDASKYG TEAELKSLIE
AFHDKNVECL ADIVINHRCA DYKDSRGVYC VFEGGTPDGR LDWGPDMICS
DDTQYSNGRG HRDTGAGFGA APDIDHLNPR VQRELTDWLN WLRTDLGFDG
WRLDFAKGYS APLARIYVDN TNPTFVVGEI WSSLIYNGDG KPSTNQDADR
PDQAVTFVDN HDTGSTQSLW PFPSDKVMQG YAYILTHPGI PCIFYDHVFD
WNLQHEIATL AEIRSRNGIH AESTLDILKA EGDIYVAMID GKVITKLGPR
YDAGGIIPSD FHVVAHGNDY CVWEKEGLRV PAGRKHY

OsRACK1A

MAGAQESLVL AGVMHGHNDV VTAIATPIDN SPFIVSSSRD KSLLVWDLTN
PVQNVGEGAG ASEYGVPFRR LTGHSHFVQD VVLSSDGQFA LSGSWDGELR
LWDLSTGVTT RRFVGHDKDV LSVAFSVDNR QIVSASRDRT IKLWNTLGEC
KYTIGGDLGG GEGHNGWVSC VRFSPNTFQP TIVSGSWDRT VKVWNLTNCK
LRCNLEGHGG YVNAVAVSPD GSLCASGGKD GVTLLWDLAE GKRLYSLDAG
SIIHSLCFSP NRYWLCAATQ DSIKIWDLES KHIVQDLKPE IPVSKNQMLY
CTSLNWSADG STLYAGYTDG TIRIYKISGF SYAG

OsTexh1

MAAEEGVVIA CHNKDEFDAQ MTKAKEAGKV VIIDFTASWC GPCRFIAPVF
AEYAKKFPGA VFLKVDVDEL KEVAEKYNVE AMPTFLFIKD GAEADKVVGA
RKDDLQNTIV KHVGATAASA SA

Supplementary Figure S3 Predictive disulfide bond in potential redox proteins

Cysteines are shown in red. Predictive disulfide bond is indicated by red line.
Signal peptide in AMY3E is shown in Bold letters.
